# Work experiences and practices of radiographers during the coronavirus disease 2019 pandemic: Future pandemic preparation

**DOI:** 10.4102/hsag.v31i0.3363

**Published:** 2026-04-24

**Authors:** Nkole Bwalya, Nape M. Phahlamohlaka, Hesta Friedrich-Nel

**Affiliations:** 1 Department of Clinical Sciences, Faculty of Health and Environmental Sciences, Central University of Technology, Bloemfontein, South Africa

**Keywords:** COVID-19, chest X-ray, radiographers, personal protective equipment, infection control, preparedness for future pandemics

## Abstract

**Background:**

During the peak of the coronavirus disease 2019 (COVID-19) pandemic, radiographers in Zambia faced heavy workloads as chest X-ray referrals outpaced staffing and the capacity of X-ray imaging equipment, and protective gear was limited. The pandemic disrupted the daily work routines of radiographers, yet little is known about how radiographers in Zambia experienced and thrived through this public health crisis.

**Aim:**

This study aimed to determine the work experiences and practices of radiographers during the COVID-19 pandemic for future reference.

**Setting:**

Two tertiary hospitals are designated as COVID-19 treatment centres in the Eastern province of Zambia.

**Methods:**

A qualitative approach with exploratory and descriptive designs was employed. A purposive sample of 11 radiographers was used for interviews to reach data saturation. The time frame for data collection was from 01 November 2023 to 30 November 2023. Data were collected through semi-structured face-to-face interviews and were analysed thematically using Braun and Clarke’s method.

**Results:**

Four themes were developed, namely: (1) personal and work-related challenges, (2) adapting to abrupt changes, (3) wellness and information support and (4) preparation for future pandemics.

**Conclusion:**

Radiographers adapted and demonstrated resilience to the high occupational pressures of patient referrals for chest X-ray imaging during the pandemic, although they were fearful of contracting the COVID-19 infection.

**Contribution:**

This article adds strategies to improve emergency X-ray imaging response and strengthen preparedness for future health emergencies, informed by radiographers’ COVID-19 experiences.

## Introduction

Zambia reported its first coronavirus disease 2019 (COVID-19) case on 18 March 2020, and by the end of the year, the numbers rose to over 20 000 (Loevinsohn et al. 2022; Phiri et al. 2021; Simulundu et al. 2021). In 2022, Zambia s population was estimated at 19.6 million people (Ahmad & Wilkins 2025; Tajik, Golzar & Noor 2024). Within this national context, the Eastern province, where Chipata District is located, recorded a population of approximately 2 454 788 in the same year (Civil Society for Poverty Reduction 2025). During the second wave of the COVID-19 pandemic, X-ray departments experienced an unprecedented increase in patient referrals for chest X-ray imaging, highlighting persistent inequities in access to X-ray imaging services in countries such as Zambia. Nevertheless, there were only two hospitals in Chipata District of Zambia’s Eastern province designated as COVID-19 centres, responsible for screening and managing patients with COVID-19. These hospitals functioned as the main referral facilities within the district, resulting in increased imaging demand, workflow reorganisation, and heightened infection prevention and control requirements during the pandemic.

Radiographers undoubtedly played a vital supporting role for physicians during the pandemic by providing the first emergency X-ray imaging services to patients with suspected or confirmed COVID-19 infection (Akudjedu et al. 2021; Stogiannos et al. 2020). They faced increased risk of exposure to COVID-19, as chest X-rays and computed tomography (CT) were central to screening and assessing the lungs of patients with suspected COVID-19 infection (Akudjedu et al. 2021; Chinene 2023; Lawal et al. 2022). The COVID-19 pandemic placed immense occupational pressure on radiographers, which was further worsened by rising infections, staff shortages and inadequate supplies of personal protective equipment (PPE) (Akudjedu et al. 2021; Naylor et al. 2022).

The experiences of Zambian radiographers mirrored those reported by other studies in the literature, with increased imaging workloads, emotional strain, fear of infection, inconsistent PPE supply, and staff shortages during the COVID-19 pandemic (Akudjedu et al. 2021; Lemon et al. 2023; Naylor et al. 2022). In South Africa, studies reported that radiographers experienced anxiety, heavy chest X-ray and CT referrals, and exhaustion from prolonged exposure to high-risk environments (Chinene 2023; Lewis & Mulla 2021; Van de Venter et al. 2021). Nevertheless, there is a gap in the literature of original research studies on the African continent, particularly in Zambia, on the impact of COVID-19 on the work of radiographers and how radiographers can prepare for future pandemics, which warranted the need for this study (Bwalya 2025).

Although the four COVID-19 waves have passed by 2025, they have left lasting personal and work experience scars on radiographers (Htun et al. 2023; Minchella et al. 2022; Puigdellívol-Sánchez et al. 2025). The uniqueness and importance of this study is in its timing, carried out in 2023, which captures the experience of radiographers from not only the first wave but also the later waves of COVID-19, when imaging demands remained high despite the interventions already put in place. Situating the study within this broader healthcare context of Zambia has the potential to address the ongoing X-ray imaging challenges and persistent gaps that extend beyond the four waves of the pandemic. The study explored the work experiences and practices of radiographers in Zambia during the COVID-19 pandemic, with the broader goal of proposing strategic plans to strengthen routine and emergency imaging responses. The insights shared in this article may help to guide radiographers and hospitals in preparing for future public health emergencies in settings similar to Zambia.

## Research methods and design

### Setting and design

This article forms part of a larger degree-related study conducted in partial fulfilment of the requirements for the Master of Radiography degree. The study was conducted at two tertiary hospitals in Chipata District, Eastern province of Zambia (Bwalya 2025), both of which were designated COVID-19 treatment centres at the peak of the pandemic in 2020. Hospital A’s bed capacity was estimated at 500, while hospital B’s bed capacity was estimated at 100. At the time of the study, both hospitals were equipped with X-ray imaging services, including mobile radiography units used widely for chest imaging of suspected COVID-19 patients. The radiographers, although in limited numbers of approximately 21 experienced a high demand for chest X-ray examinations during the COVID-19 pandemic. This study adopted a qualitative approach with exploratory and descriptive research designs because these designs are well-suited for uncovering insights into radiographers’ experiences and work practices during the COVID-19 pandemic. Such an approach enabled the researchers to explore an understudied phenomenon and describe it within its real-world context (Creswell & Poth 2018; Saunders, Lewis & Thornhill 2023).

### Population and sampling

The target population for the study comprised all 21 radiographers across the two selected hospitals. Using purposive sampling, radiographers were recruited to participate voluntarily in face-to-face interviews, with 11 participants included once information redundancy was reached (Bwalya 2025). Furthermore, following preliminary data analysis, a panel of experts comprising six diagnostic radiographers was purposely invited to provide inputs aimed at strengthening and refining the strategies proposed by the researchers in this study. The panel reviewed the draft strategies derived from the interview findings and provided feedback through structured discussions, which informed the final recommendations. Purposive sampling was used because it allowed the researcher to deliberately select participants who possess specific knowledge, experience or characteristics relevant to the study (Ahmad & Wilkins 2025; Tajik et al. 2024).

#### Exclusion and inclusion criteria

Radiographers performing both CT and general radiography who were employed at the selected hospitals during the COVID-19 pandemic and who provided informed consent were eligible to participate in the interviews. Sonographers, nuclear medicine radiographers, and therapeutic radiographers were excluded from the study because of the fact that the study focused specifically on professionals who perform routine general X-ray and CT imaging for diagnosing and management of COVID-19.

### Data collection procedure

An interview guide was developed based on relevant literature and the study objectives. The guide was piloted with two radiographers who met the inclusion criteria but was not included in the final sample. Feedback from the pilot interview informed minor refinements to question wording to enhance clarity and relevance prior to formal data collection.

Following the pilot, the researcher visited two research sites and invited participants after introducing the study and its objectives in the meeting. Thereafter, the participants who were available and willing to take part in the study were given an information letter and an informed consent form to sign. The interviews were conducted in a private room at each hospital, each of the 11 interviews lasting approximately 30 min. No incentives were provided to the participants. The central research question that opened the interviews was: *What were your work experiences as a radiographer during the COVID-19 pandemic?* The probing questions used during the interviews included the following:

- *What practices did you adopt during the COVID-19 pandemic in the X-ray departments?*
- *What kind of support have you received from hospital management to help you cope with the COVID-19 pandemic?*
- *What strategies would you recommend to better prepare X-ray departments for future pandemics demanding X-ray imaging intervention?*

Data saturation was determined after the ninth interview, where no new codes were identified from the experiences shared by the participants; therefore, the two subsequent interviews yielded no new information to answer the research question (Alordiah & Oji 2024; Fusch & Ness 2015; LaDonna, Artino & Balmer 2021). All the interviews were audio-recorded with participants’ consent, and field notes were taken to capture nonverbal cues and contextual observations.

### Data analysis

The recorded interviews were transcribed verbatim within 72 h by an independent transcriber who signed a confidentiality agreement to maintain confidentiality. The authors then conducted data analysis using Braun and Clarke’s six-step thematic analysis approach, which involved familiarisation with the data, coding, developing initial themes, reviewing and refining these themes, naming them, and producing the final report (Braun & Clarke 2021; Naeem et al. 2023). Field notes were used to supplement the interview data by capturing contextual observations and non-verbal cues, which helped the researchers to understand the meanings of the participants’ experiences. The interview transcripts were imported into ATLAS.ti 2024, where the data were coded and categories of codes were developed. An open coding approach was employed to generate codes, which were then organised into related categories. Before uploading the transcripts into ATLAS.ti 2024, for coding, all identifying information was removed to protect the anonymity of the participants and the hospitals involved. The theme and subthemes were developed based on the categories of codes in ATLAS.ti (Braun & Clarke 2021; Naeem et al. 2023; Williams & Moser 2019).

### Trustworthiness

Credibility, dependability, confirmability and transferability are important criteria for applying trustworthiness in qualitative research (see Table 1). Credibility ensures that findings are believable and accurate, and provide a realistic report of participants’ experiences (Lincoln & Guba 1985; Shenton 2004). Dependability addresses the stability and consistency of the research process over time, thereby suggesting that similar results could be achieved under comparable settings (Denzin et al. 2024; Haq Kaka et al. 2023). Confirmability highlights the extent to which findings are shaped by participants rather than researcher bias or interest (Denzin et al. 2024; Lincoln & Guba 1985), while transferability defines the applicability of research findings to other settings with similar characteristics (see Table 1) (Drisko 2025; Lincoln & Guba 1985; Stalmeijer, Brown & O’Brien 2024).

**TABLE 1:** Measures applied to ensure trustworthiness.

Criterion	Strategy
Credibility	Direct quotations from participants are included in the findings to support the authenticity of their voices. Interview data were cross-referenced with field notes to strengthen credibility and reduce bias. Strategies proposed in the article were consulted with a panel of five diagnostic radiographers via a virtual meeting.
Dependability	Audit trail of research methods and data analysis is provided. Interview scripts and field notes are securely stored on the CUT OneDrive for a period of 5 years.
Confirmability	Developed themes were shared with participants to verify accuracy in interpreting their experiences. Participants’ feedback was incorporated to refine themes and ensure confirmability.
Reflexivit	Researchers set aside personal biases to maintain neutrality and keep the focus on participants’ views during analysis and reporting. As a radiographer, the interviewer used his professional insight to enrich the interviews while bracketing his views to avoid influencing data collection or analysis.
Transferability	A detailed account of the methodology is provided to aid the readers in judging the applicability of findings to their respective contexts. Participants were selected based on inclusion criteria to ensure relevant representation for the study context.

*Source*: Creswell, J.W. & Creswell, J.D., 2017, *Research design: Qualitative, quantitative, and mixed methods approaches*, 5th edn., SAGE Publications, Thousand Oaks, CA; Denscombe, M., 2021, *The good research guide: Research methods for small-scale social research*, 7th edn., McGraw-Hill Education, Open University Press; Lincoln, Y.S. & Guba, E.G., 1985, *Naturalistic inquiry*, 1st edn., SAGE Publications; Polit, D.F. & Beck, C.T., 2021, *Essentials of nursing research: Appraising evidence for nursing practice*, 10th edn., Wolters Kluwer, Philadelphia, PA; Tavakol, M. & Sandars, J., 2014, ‘Quantitative and qualitative methods in medical education research: AMEE guide No 90: Part II’, *Medical Teacher* 36(10), 838–848. https://doi.org/10.3109/0142159X.2014.915297

CUT, Central University of Technology, Free State.

### Ethical considerations

Ethical clearance for the study was granted in 2023 by the Lusaka Apex Medical University Biomedical Research Ethics Committee (LAMUBREC). The ethical clearance number is 00463-23. Data collection commenced on 01 November 2023 following receipt of ethics approval and gatekeeper permissions from the participating hospitals earlier that month. The researcher obtained informed consent from all participants for both participation in the study and audio recording of their interviews. The recordings were transcribed by an independent professional who signed a confidentiality agreement and agreed to delete the recordings upon completing the transcriptions. To ensure data security and protect participant privacy, all transcribed files were stored on a password-protected laptop and cloud storage system at the Central University of Technology, Free State. This research study was conducted in accordance with the principles outlined in the Declaration of Helsinki (1975, revised in 2013) (Shrestha & Dunn 2020). Participants were informed that they could withdraw from the interview at any time without any consequence. Contact details for professional support services were provided in case of distress.

## Results

A total of 11 participants were interviewed, comprising six men and five women. All participants were recruited from public hospitals, as only these facilities were designated as COVID-19 treatment centres during the pandemic. Their ages ranged from 20 to 49 years, with work experience varying between 2 and 20 years. In terms of qualifications, two participants held Bachelor’s degrees, while the remaining nine possessed diploma-level qualifications. Data analysis yielded four overarching themes, each with associated subthemes (Bwalya 2025) (see Table 2).

**TABLE 2:** Themes and subthemes.

Themes	Subthemes
1. Personal and work-related challenges	1.1 Mental and emotional distress 1.2 Workload, staffing and resources
2. Adaptation to abrupt changes	2.1 Adjustment of routine work practices and schedules 2.2 Infection prevention and control measures 2.3 Screening and triaging patients
3. Wellness and information support	3.1 Mental health support care 3.2 In-service training and information sessions
4. Preparation for future pandemics	4.1 Ongoing training and planning 4.2 Strengthening support for investment in teleradiology

### Theme 1: Personal and work-related challenges

This theme captures the acute psychological, emotional, trauma, anxiety, fear and occupational difficulties participants faced during the COVID-19 pandemic (Bwalya 2025). The radiographers reported having to manage increased workloads, staff shortages, severe resource constraints, particularly during the peaks of the pandemic, and the possibility of infecting their families with COVID-19. These feelings were worsened by witnessing colleagues falling ill or dying, which led to occupational stress and mental fatigue. These challenges were presented across the following themes.

#### Subtheme 1.1: Mental and emotional distress

Participants reported experiencing anxiety, fear and emotional strain while working during the pandemic. Exposure to very sick patients, fear of contracting COVID-19, and concern about transmitting the virus to family members negatively affected their mental well-being. These experiences were further exacerbated by seeing workmates become sick or die, leading to mental and emotional distress:

‘It became a challenge, filled with anxiety and fear during the pandemic.’ (Participant 4, Male, 22 years)

‘Dealing with critically ill patients and potential exposures to the virus itself had a toll on us health workers.’ (Participant 11, Female, 29 years)

#### Subtheme 1.2: Workload, staffing and resources

Participants also narrated how the increase in infections led to a steady increase in the demand for chest X-rays and CT scans, which contributed to heavier workloads and a heightened need for additional staff and PPE. Moreover, anxiety and fear of contracting the virus contributed to staff shortages because employees who tested positive were required to take sick leave and self-quarantine at home, thereby temporarily reducing the available workforce (Bwalya 2025):

‘Yes, and then also, you find that someone tested COVID-positive. So, it meant that they can’t work; they have to go into self-quarantine.’ (Participant 1, Male, 45 years)

‘The hospital experienced a staff shortage due to the increased number of patients. Then, some of the staff received a diagnosis of COVID-19.’ (Participant 3, Male, 27 years)

‘It was quite a stressful period because there was an increase in the workload, because several chest X-ray examinations were requested.’ (Participant 5, Male, 24 years)

### Theme 2: Adaptation to abrupt changes

This theme relates to the way radiographers rapidly adjusted their clinical practices, work schedules and infection control measures to respond to the unprecedented demands of the COVID-19 pandemic and minimise risk of infection. The report also addresses how triaging was implemented. The following subtheme highlights these changes.

#### Subtheme 2.1: Adjustment of routine work practices and schedules

Furthermore, radiographers modified standard imaging protocols to accommodate the clinical condition of patients during this period, because many patients were very sick, immobile or referred on stretchers. This positioning decision was therefore guided by patient condition and infection control considerations rather than diagnostic preference. One participant indicated that radiographers adjusted exposure settings and imaging approaches during chest X-ray examinations to optimise diagnostic quality on the first attempt, thereby reducing the need for repeat imaging and minimising cross-contamination. X-ray departments implemented a range of strategies to minimise exposure risk, including physical space modifications and stringent PPE usage during X-ray examinations. Participants adhered to physical distancing as prescribed by World Health Organization guidelines by making changes to the waiting areas. Another strategy was to adjust shifts in the X-ray department - introducing shorter shifts and reducing the number of shifts per week - to handle the heavy workload during the pandemic. For example, under the revised schedule, participants typically worked four shifts per week, with each shift running for five hours, for example a shift would run-from 13:00 to 18:00, allowing them to spend less time in the hospital environment:

‘Yes. And the exposure techniques and imaging angles were made to sort of optimise diagnostic quality while reducing the risk of cross-contamination.’ (Participant 11, Female, 29 years)

‘During that period, we were working maybe mainly four per shift. So, our shifts were from 13 to 18. Yes, so mainly four because of the huge numbers.’ (Participant 1, Male, 45 years)

‘You must ensure the imaging plate or detector is clean. If not, use a plastic barrier that can be removed and the plate cleaned after exposure to prevent infection spread.’ (Participant 2, Male, 38 years).

‘We have some social distancing practices that were practised by the department.’ (Participant 10, Female, 25 years)

#### Subtheme 2.2: Infection prevention and control measures

Participants described how existing infection prevention and control measures (IPC) practices were strengthened and more rigorously applied, including enhanced patient triage and stricter adherence during X-ray examinations, to minimise cross-infection between staff and patients. Radiographers also minimised patient movements during imaging to reduce the risk of cross-infection and accommodate patients who were critically ill or unable to stand. Supine positioning was frequently used for chest X-rays, particularly for patients on stretchers or with limited mobility:

‘So, we had to focus on the infection control measures, which was like cleaning, disinfecting, like we would make sure the room is well disinfected before we attend to the patient.’ (Participant 6, Male, 30 years)

‘Yes, the radiographers minimised patients’ movements by using a supine position, particularly for chest X-rays.’ (Participant 3, Male, 27 years)

#### Subtheme 2.3: Screening and triaging patients

Radiographers described implementing new practices, including patient triage and stricter PPE usage, to manage the increased risk of cross-infection during the pandemic. Triaging was conducted primarily to prioritise patients for imaging based on clinical urgency and to optimise limited resources, including PPE, while maintaining infection prevention protocols. The participants, as frontline health workers, rearranged the work environment by building on the triage of patients and adhering to COVID-19 safety protocols. They identified cases requiring triage, prioritising the immediate treatment of critically ill patients over others (Bwalya 2025). Radiographers also collaborated with other healthcare professionals to screen patients for symptoms of the virus and evaluated walk-in patients for symptoms such as headaches or flu-related COVID-19. Furthermore, when patients with COVID-19 infections needed to go to the X-ray department, specially developed, dedicated routes were used to keep them separate from other patients:

‘We adopted a new culture that we are not used to triaging of patients and PPE usage.’ (Participant 2, Male, 38 years)

‘So, like the timetable, it had to change, you find that in a day, maybe per shift, there’ll be only one person who’ll be working for us not to get COVID-19.’ (Participant 9, Female, 22 years)

‘To lower the risk of transmission, we implemented these distinct pathways and workflows to separate COVID-19 and non-COVID-19 patients, ensuring everyone’s safety.’ (Participant 3, Male, 27 years)

‘We were also involved in patient screening for COVID symptoms. Yes, the triage tables used to involve maybe a radiographer.’ (Participant 1, Male, 45 years)

‘Radiographers requested to be in triage for portable procedures to screen patients who were suspected of the virus and confirm cases of patients who actually had the COVID-19 symptoms.’ (Participant 5, Male, 24 years)

### Theme 3: Wellness and information support

This theme highlights the essential role played by hospital management in supporting radiographers and other frontline healthcare workers during the COVID-19 pandemic. Management interventions extended beyond maintaining operations to protecting staff health, safety and well-being (Bwalya 2025). The following subthemes highlight how radiographers’ wellness and information support was implemented.

#### Subtheme 3.1: Mental health support care

Radiographers reported that when staff became ill, management provided medication, allowed time off for recovery, and offered flexible shifts. Counselling services, stress-management programmes and other mental health resources were introduced to support frontline workers:

‘Yes, the hospital used to give us vitamin C and azithromycin and encouraged us to stay hydrated by drinking plenty of water.’ (Participant 1, Male, 45 years)

‘Management ensured that they supported us emotionally, and caring for the carer was enhanced during the pandemic.’ (Participant 2, Male, 38 years)

#### Subtheme 3.2: In-service training and information sessions

Participants also received training and information sessions on COVID-19 and infection control, while regular meetings and internal communication channels kept staff updated on new cases, treatment guidelines and related developments:

‘The management actually provided some training, adequate training to educate every member of staff on infection control measures and protocols to follow.’ (Participant 5, Male, 24 years)

‘Management provided mental health support as well for the clinicians and the officers attending to patients in the diagnostic centres.’ (Participant 10, Female, 25 years)

‘Yeah, we had to participate in additional training and educational programmes related to COVID-19 imaging protocols, the safety measures and the use of other technologies.’ (Participant 11, Female, 29 years)

### Theme 4: Preparation for future pandemics

This theme addresses participants’ reflections on how X-ray departments should position themselves to respond to future health crises. Participants highlighted experiences from the COVID-19 pandemic informed a proactive approach to preparedness. Preparation was thus seen as an ongoing, multifaceted process aimed at ensuring the resilience and readiness of X-ray imaging services in the face of emerging health challenges (Bwalya 2025). The following subthemes present these reflections.

#### Subtheme 4.1: Ongoing training and planning

Radiographers highlighted the need for continuous staff training, strategic workforce planning, and adherence to established policy frameworks:

‘Continuous research must guide how we improve our pandemic response and maintain rapid response teams even before outbreaks occur.’ (Participant 1, Male, 45 years)

‘This should actually include some staff and resource allocation, adequate training, and education for control protocols and staff equipment training, some clear communication, flexible scheduling should also be part of it to consider the well-being and physical and just mental well-being of our radiographers.’ (Participant 5, Male, 24 years)

#### Subtheme 4.2: Strengthening support for investment in teleradiology

Radiographers highlighted the need for the adoption of teleradiology in the face of infections, such as COVID-19, to help limit cross-infection while ensuring that only a few radiographers work in the clinical area at a time to minimise exposure to radiographers:

‘So, the first thing I would suggest as a policy by the government for future pandemics is that at least only a few staff should be in clinical areas. Okay. When there is a pandemic, even a staff is a potential carrier of the infection.’ (Participant 2, Male, 38 years)

‘They should invest in telemedicine and remote consultation. People should not be physically present in one place.’ (Participant 8, Female, 27 years)

### Development of strategies to support emergency X-ray imaging response for the future

Strategies to support continuity of X-ray imaging during future pandemics were developed based on the study’s findings and relevant COVID-19 literature. A panel of six radiography experts, each with at least 5 years of clinical or academic experience and holding a master’s or PhD, was purposively selected to review the draft strategies. Eligible participants received formal invitations outlining the study’s purpose, session logistics and ethical considerations. A virtual review session was held on 26 June 2025 via Microsoft Teams, during which the principal researcher presented the study’s objectives, key findings and draft strategies. Participants provided implied informed consent by voluntarily agreeing to participate after being fully informed about the study. Expert feedback was collected immediately afterwards using a structured Google Forms survey to assess clarity, relevance, applicability and validity of the proposed strategies.

## Discussion

This study aimed to determine the work experiences and practices of radiographers during the COVID-19 pandemic for future reference. Although the COVID-19 pandemic has passed, this study offers important insights for future radiographers by documenting the experiences and lessons of those who worked on the frontline in Zambia. These perspectives, captured from radiographers who practised during the pandemic, provide valuable guidance for strengthening preparedness and radiography practice in future health emergencies. The study participants’ accounts of experiencing mental, emotional and operational challenges during the pandemic reflect findings by Evanoff et al., Lee et al. & Sims et al. regarding experiences of radiographers during the COVID-19 pandemic (Evanoff et al. 2020; Lee, Jang & Kim 2023; Sims et al. 2022). Fear of infection resulted in anxiety and emotional exhaustion, which was worsened by witnessing colleagues falling ill or dying (Alwesmi, Dator & Karavasileiadou 2022; Anderson-Shaw & Zar 2020; Chinene 2023). Furthermore, the greater demand for imaging, particularly for chest X-rays and CT scans, caused overwhelming workloads. Staff shortages attributed to illness and quarantine forced radiographers to work extended hours, which led to fatigue and burnout. Furthermore, these challenges were attributed to inadequate PPE and imaging supplies (Akudjedu et al. 2021; Konstantinidis 2024; Naylor et al. 2022).

New infection prevention protocols, for instance, strict PPE usage, physical distancing and disinfection, necessitated adaptation by participants. Literature highlights similar findings detailing how radiographers during the pandemic adapted to maintain the continuity of services while embracing infection prevention protocols (Becker et al. 2021; Cobb et al. 2021; Hughes et al. 2023; Phan et al. 2019). Routine chest X-ray projections were disrupted and replaced by emergency protocols, requiring radiographers to tailor patient care to accommodate the patient’s condition. Key changes included the increased use of supine chest X-ray projections for non-ambulatory patients and the increased use of mobile radiography to reduce cross-infection. These practices align with research studies conducted in America and Australia, highlighting that chest X-rays and chest CT scanning are essential in the identification of pulmonary infections, such as COVID-19 and its complications (Martínez Chamorro et al. 2021; Serena Low et al. 2021). Although erect chest X-rays are preferred, supine imaging was sometimes used for critically ill or immobile patients to minimise movement, reduce infection risk, and accommodate oxygen or ventilator support, influencing radiographers’ practices during the COVID-19 pandemic.

Participants adjusted exposure factors and imaging techniques to optimise diagnostic quality, reduce repeat examinations and minimise contact time, while adhering to the as low as reasonably achievable (ALARA) principle during chest radiography. However, specific exposure factor adjustments were not detailed by participants, and the study does not quantify exact kVp or mAs settings used. Careful optimisation of patient X-ray exposure remains pivotal for producing diagnostic-quality images to support the detection of lung infections (Chan et al. 2022; Steuwe et al. 2020; Tay et al. 2021; Varghese et al. 2024).

Participants relied on simple strategies to manage continued X-ray imaging services, such as reduced working hours, to counteract staff and resource constraints. Similar challenges related to equipment limitations during the COVID-19 pandemic have been reported in the Zambian context in previous studies (Bwanga et al. 2023; Mbewe et al. 2020; Ng’andwe & Bwanga 2022). First-world countries benefited from modern imaging infrastructure, with mobile digital radiography reducing patient transfers, picture archiving and communication system (PACS) and automated workflows sustaining efficiency, and optimised digital capacity ensuring resilience (Mossa-Basha et al. 2020; Society of Radiographers 2020). Globally, evidence indicates that advanced imaging systems, remote reporting, and rapid procurement mechanisms supported the continuity of imaging services in well-resourced settings (Naylor et al. 2022; Omboni et al. 2022).

The triaging of patients was perceived by participants as an important strategy for reducing potential disease transmission within the hospital. In addition, participants expanded their roles to include patient screening and triaging, which reflected increased interdisciplinary collaboration between radiographers and other healthcare professionals. While patient triaging is not traditionally central to radiographers’ routine scope of practice, the pandemic necessitated adaptive role extension to support infection prevention and workflow efficiency. Similarly, Stogiannos et al. (2020) highlight in their study that radiographers have a vital role in the triaging of suspected and known COVID-19 patients during pandemics (Becker et al. 2021).

Hospital management provided emotional and mental support during the pandemic. Participants emphasised their need for accessible counselling and stress management services; this echoes findings from other sub-Saharan African contexts where such interventions, although limited, have had positive outcomes (Cobb et al. 2021; Martínez Chamorro et al. 2021). Ensuring staff have sufficient rest between shifts enhances their well-being, which subsequently strengthens the delivery of high-quality healthcare services, particularly in high-volume settings such as X-ray departments during the pandemic (De Lima Garcia et al. 2019; Kumar et al. 2023).

To prepare for future pandemics, participants highlighted the importance of proactive strategies, including continuous infection control training, clear staffing protocols and access to mental health support. They also highlighted the need for structured policies, interdisciplinary collaboration among health professionals in the hospital and investment in teleradiology to enhance remote care and reporting. Proactive and well-formulated strategies such as continuous infection control training, access to mental health support and improved staffing protocols play an important role in providing quality healthcare during the pandemic (Ng’andwe & Bwanga 2022; Varghese et al. 2024). The work experiences and practices of radiographers in Chipata District of Zambia during the COVID-19 pandemic align closely with those reported in other sub-Saharan African countries (Akudjedu et al. 2021; Dramowski et al. 2020; Ezema et al. 2023).

### Recommendations

The authors drew on radiographers’ work experiences and practices during the COVID-19 pandemic, along with their suggestions for future preparedness, to synthesise strategies that ensure the continuity of X-ray imaging services in the event of a similar health crisis. These strategies are intended not only to support radiographers in maintaining essential imaging services but also to advise health authorities on strengthening the readiness of X-ray departments to respond effectively to future public health emergencies requiring imaging interventions (see Table 3).

**TABLE 3:** Strategies to support emergency X-ray imaging response for the future.

Strategy	Focus area
1. Enhance radiography education and training on emergency imaging	Public health emergency response Simulations and emergency drills
2. Review and update hospital procedures on disaster management	Infection prevention and control Resource allocation and procurement
3. Establish a wellness clinic for staff	Enhance mental health support services Awareness and engagement
4. Plan and budget for public health emergency response	Investment in X-ray imaging facilities Emergency response funding

### Strategy 1: Enhance radiography education and training on emergency imaging

To improve the responsiveness of radiography services during pandemics, radiographers must be equipped with specialised knowledge and skills through targeted education. This strategy proposes the introduction of workshops or short courses about emergency imaging, infection prevention and control and patient triage, as well as enhancing interprofessional education for radiographers to enable them to work as part of a multidisciplinary team with other healthcare professionals. The proposed workshops should be practice-oriented and use case-based learning and simulations. The responsibility of introducing these workshops should be carried by training institutions and professional bodies such as the Radiological Society of Zambia and the Society of Radiographers of South Africa. This guideline proposes that hospital management implement periodic emergency drills to assess staff preparedness for responding to public health emergencies.

### Strategy 2: Review and update hospital procedures on disaster management

To prepare for continuity and safe X-ray imaging services during pandemics, hospitals should regularly review and update their public health emergency response policies. These reviews should include alignment of infection prevention and control protocols with current national guidelines and World Health Organization standards. Radiographers, as frontline imaging professionals, rely on clearly defined, department-specific procedures, such as prescriptions for appropriate PPE usage, equipment disinfection and staff scheduling to minimise infection risks while maintaining service delivery.

Radiographers’ active participation in the development and review of emergency policies is crucial not only to strengthen their knowledge of outbreak protocols but also to affirm their vital role as frontline responders. Because they are directly involved in patient screening through chest X-ray imaging, engaging radiographers in policy processes enhances their role clarity, accountability and recognition within multidisciplinary emergency response teams. Hospitals are therefore encouraged to establish clear institutional emergency plans that explicitly incorporate X-ray imaging services. For instance, each facility’s emergency response task team should be structured and readily activated to address any unforeseen public health crisis.

### Strategy 3: Establish a wellness clinic for staff

To enhance mental health support, the authors propose that a dedicated wellness centre for frontline healthcare professionals be established outside the premises of the public hospital where they work, to ensure their freedom and comfort. A wellness centre should offer structured, confidential and accessible mental health services, such as trauma and psychological counselling and stress relief programmes, facilitated by trained personnel such as social workers and clinical psychologists. This guideline is supported by the insights of Evanoff et al. (2020) and Mosadeghrad et al. (2024), which emphasise the value of mental health support that addresses the well-being of healthcare professionals during public health emergencies.

The hospital wellness clinic should have ongoing awareness and engagement initiatives to promote a supportive, stigma-free work culture that is free of any form of discrimination or violence, as noted during COVID-19. Appointing radiographers as wellness champions will encourage peer-led support, enhance mental health advocacy and foster a culture of inclusivity and resilience in radiography departments. Over time, these efforts are seen to have the potential to contribute to higher job satisfaction, reduced absenteeism and a more emotionally resilient X-ray workforce.

### Strategy 4: Plan and budget for public health emergency response

The national health ministry should prioritise dedicated emergency response funding in reserves to enable the swift acquisition of essential resources during public health emergencies. These funds should support the procurement of consumables, doing short-term infrastructure upgrades and hiring additional administrative staff and radiographers to meet increased demands for patient care and support services. Simultaneously, the ministry should invest in upgrading radiology infrastructure to enhance the capacity of imaging departments to handle unprecedented requests for X-ray examinations. Key interventions include expanding imaging rooms to meet safety standards, procuring adequate portable X-ray units for mobile radiography, installing reliable backup power systems to ensure uninterrupted operations and transitioning to digital technologies such as picture archiving and communication systems to facilitate teleradiology and remote image interpretation.

### Strengths and limitations

A key strength of this study is that one-on-one interviews enabled the collection of rich, personal accounts from radiographers who worked during the pandemic. However, the study was limited to diagnostic radiographers from two major hospitals in Zambia’s Eastern province. As is characteristic of qualitative research, the findings are not intended to be statistically generalisable but may be transferable to contexts with similar health system structures, resource constraints and professional practices. The reliance on self-reported interview data also introduces potential bias, but the interviewer’s positionality has been clarified in Table 1.

## Conclusion

This study showed that radiographers continued providing essential chest X-ray imaging despite overwhelming referrals, staff shortages, limited resources including shortages of PPE and constraints related to imaging consumables and the fear of COVID-19 infection. Their resilience and adaptive practices reflect their role as frontline healthcare professionals, navigating high-risk situations and continuing essential services during the pandemic. These experiences emphasise the need for stronger infection control training, mental health support and investment in telemedicine for remote reporting and sharing of X-ray images to improve preparedness for future health emergencies. Finally, this study synthesised strategic measures to strengthen emergency patient imaging and support radiographers’ well-being, offering insights applicable to other African contexts.
